# Biomanufacture of nano-Pd(0) by *Escherichia coli* and electrochemical activity of bio-Pd(0) made at the expense of H_2_ and formate as electron donors

**DOI:** 10.1007/s10529-016-2183-3

**Published:** 2016-08-08

**Authors:** J. Courtney, K. Deplanche, N. V. Rees, L. E. Macaskie

**Affiliations:** 1 School of Biosciences, University of Birmingham, Edgbaston, Birmingham, B15 2TT UK; 2 School of Chemistry, University of East Anglia, Norwich Research Park, Norwich, Norfolk NR4 7TJ UK; 3 Finovatis, 68 Cours Lafayette, 69003 Lyon, France

**Keywords:** Bio-Pd, *E. coli*, Electrochemical activity, Fuel cell, Hydrogen production, Palladium

## Abstract

**Objective:**

Palladised cells of *Desulfovibrio desulfuricans* and *Shewanella oneidensis* have been reported as fuel cell electrocatalysts but growth at scale may be unattractive/costly; we have evaluated the potential of using *E. coli*, using H_2_/formate for Pd-nanoparticle manufacture.

**Results:**

Using ‘bio-Pd’ made under H_2_ (20 wt%) cyclic voltammograms suggested electrochemical activity of bio-NPs in a native state, attributed to proton adsorption/desorption. Bio-Pd prepared using formate as the electron donor gave smaller, well separated NPs; this material showed no electrochemical properties, and hence little potential for fuel cell use using a simple preparation technique. Bio-Pd on *S. oneidensis* gave similar results to those obtained using *E. coli.*

**Conclusion:**

Bio-Pd is sufficiently conductive to make an *E. coli*-derived electrochemically active material on intact, unprocessed bacterial cells if prepared at the expense of H_2_, showing potential for fuel cell applications using a simple one-step preparation method.

## Introduction

A proton electrolyte membrane fuel cell (PEM-FC) comprises anode and cathode catalysts separated by a proton exchange membrane. Catalytic splitting of H_2_ anodically provides electrons, which recombine with protons and atmospheric O_2_ at the cathode, forming water. Alternatively, microbial fuel cells can generate electricity from waste (e.g. Wu et al. 2014; Sanchez et al. 2015) but the low power density limits their use (Jang et al. 2013). Bacteria can make H_2_ from waste to supply the anodic reaction (Macaskie et al. 2005; Redwood et al. 2012); residual bacteria can then make metallised FC-catalyst biomaterial with precious metals biorecovered from waste (Orozco et al. 2010).

Yeast (Dimitriadis et al. 2007) or bacterial (Yong et al. 2007) cells support palladium nanoparticle (Pd-NP) PEM-FC electrodes; the biomaterial required sintering (carbonisation) to improve conductivity (Yong et al. 2007). The highest power output, comparable to commercial FC catalyst, used sintered, platinised cells of *Desulfovibrio desulfuricans,* (Yong et al. 2007) but palladised sintered cells of *D. desulfuricans* and also *E. coli* (bio-Pd_*D. desulfuricans*_ and bio-Pd_*E. coli*_) were also active (Yong et al. 2010).

Later work showed electrochemical activity of palladised native cells of *D. desulfuricans*, increased by adding formate (electron donor) to live, but not heat-killed cells while lactate supported activity using bio-Pd on live cells only; cytochromes and periplasmic hydrogenases were implicated (Wu et al. 2011). Similarly, Ogi et al. (2010) used Pd-NPs on cells of *Shewanella oneidensis* in a PEM-FC to give a power output of 90 % of that of a commercial catalyst at a Pd loading of 20 % of the biomass dry weight. Studies have focused on the anodic reaction whereas the rate-limiting cathodic O_2_ reduction reaction (ORR) is relatively unexplored. Non-metallised, active cells were used cathodically, with limited success (Jang et al. 2013). Williams (2016) achieved the ORR by using bio-Pt_*E. coli*_, comparably to a commercial FC catalyst, following chemical stripping of biochemical components to unmask an electrochemically-responsive Pt surface (Attard et al. 2012).

Substitution of Pd into the electrodes would offer major cost benefits. A PEM-FC with a ‘bio-Pd’ anode (Yong et al. 2007, 2010) gave consistent power output over several weeks although Pd is generally held to have a short catalyst life; durability targets are 5000 h of operation for automotive application and 40,000 h for stationary FCs over 10 years (Rice et al. 2015).

*D. desulfuricans* is not readily scalable; it is difficult to grow to high cell densities, while the removal of H_2_S (a powerful catalyst poison) is required; this restriction could also apply to *S. oneidensis* which produces H_2_S from various sulfur compounds (Wu et al. 2015). Practically, one could use an organism grown aerobically to high cell densities (e.g. waste bacteria from other applications). Hydrogenases, [which make bio-Pd (Deplanche et al. 2010)], are then upregulated during anaerobic resuspension for catalyst manufacture (Zhu et al. 2016).

Yong et al. (2007, 2010) and Ogi et al. (2011) agreed that maximum FC-activity requires a high loading of Pd(0) (20 wt%). Native palladised cells of *D. desulfuricans* were active in electron transport (Wu et al. 2011) but the Pd(0)-loading was not stated; it may be possible to use native bio-Pd_*E. coli*_ electrocatalytically, given suitable conductivity. This study compared bio-Pd_*E. coli*_ in two ways using voltammetry to assess electrochemical activity. Successful proof would open the harnessing of ‘2nd life’ *E. coli* biomass into new catalysts, using these bacteria to make FC catalysts from metallic wastes (above) as well as potentially harnessing the tools of synthetic biology towards ‘designer’ catalysts, since *E. coli* is the ubiquitous ‘workhorse’ organism for molecular engineering.

## Materials and methods

### Cell culture

*E.coli* MC4100 was as described previously (Deplanche et al. 2010). Cultures were grown anaerobically in sealed bottles in lysogeny broth (LB) (10 g tryptone/l, 5 g yeast extract/l, 10 g NaCl/l). Cultures were harvested by centrifugation in the mid-logarithmic phase of growth (OD_600_ of 0.5–0.7), washed three times in 100 ml 20 mM MOPS/NaOH buffer (degassed, pH 7.2) and resuspended in a small volume of the same buffer (4 °C) until use, usually the next day. Cell concentration (mg/ml) was determined by reference to a pre-determined OD_600_ to dry weight conversion. Some tests used *Shewanella oneidensis* strain MR1 grown and prepared in the same way.

### Palladium mineralisation

To make bio-Pd, 25 ml concentrated resting cell suspension was transferred under O_2_-free N_2_ into 200 ml serum bottles and 40 ml degassed 10 mM Pd(II) [sodium tetrachloropalladate (Na_2_PdCl_4_ in 0.01 M nitric acid, aq.)], or palladium chloride (PdCl_2_ in 0.01 M HNO_3_) was added to give a final loading of 20 % (w/w) Pd on biomass. Pd/cells were left to stand (30 min, 30 °C) with occasional shaking to promote biosorption of Pd(II) complexes before either H_2_ was sparged through the suspension (200 ml/min, 20 min) or 5 ml 50 mM sodium formate (degassed) was added to reduce cell surface-bound Pd(II) to Pd(0). To confirm the metal content of the metal/cell catalysts, the residual free Pd(II) ion content in solution was monitored at all stages of the preparation spectrophotometrically using the tin chloride method (Deplanche et al. 2010).

### Electron microscopy

For scanning transmission electron microscopy (STEM), the Pd cell samples were immersed in 2.5 % glutaraldehyde (1 day). Secondary fixation used 1 % OsO_4_ (1 h; omitted where analysis was to be done using energy dispersive X-ray microanalysis (EDS)). Samples were dehydrated using 50, 70, 90 and 100 % ethanol (2 × 15 min for each). Two further dehydration steps (15 min) were made in propylene oxide and samples were embedded in a 1:1 mixture of propylene oxide/resin (45 min; gentle shaking) then pure resin (1 h, then *in vacuuo*; 30 min) and cured (60 °C; >16 h; atmospheric pressure). For STEM, samples were cut into thin sections (diamond knife; 50–150 nm) and collected on electron microscope girds (Formvar film/carbon coated). Standard stains of uranyl acetate and lead citrate were added and the samples were examined using a Jeol JSM-7000f FE-SEM with an Oxford Inca EDS detector.

### Electrochemical analysis of bio-Pd**(0)** prepared under H_2_ or with formate

Working electrodes were made using the drop-cast technique. A 3 mm (EDI101, Radiometer Analytical, Salford, UK) and 5 mm diameter (afe2m050gc, Pine Research Instrumentation, Durham) glassy carbon rotating disc electrode (RDE) tip was taken and 20 μl (5 mm) or 10 μl (3 mm) of bio-Pd suspension was deposited onto a polished tip. The tip was covered with a beaker and left to dry (12 h). A three electrode half-cell set-up was used with a surrounding water jacket (25 °C). The reference electrode (RE) was a normal H_2_ electrode (NHE), the counter electrode (CE) was platinum gauze and the working electrode (WE) was as above. The WE was connected to an Autolab (PGStat302N, Metrohm-Autolab, Utrecht, The Netherlands) potentiostat/galvanostat through either a EDI101 RDE (Radiometer Analytical, 3 mm) or a Pine modulated speed rotator (AFMSRCE, Pine Research Instrumentation, 5 mm). The electrolyte was 0.1 M perchloric acid (Sigma-Aldrich, *Trace*SELECT Ultra), with a N_2_ purge (20 min). Prior to electrochemical analysis the electrode was ‘cleaned’ by cycling the applied potential between 0 and 1.1 V (vs. NHE) (50 cycles at 0.1 V/s). The WE was then placed in the half-cell described above and cyclic voltammetry (CV) data was recorded by applying potential scans between 0 and 1.1 V (vs. HE) at various scan rates (0.1, 0.5, 0.05, 0.025 V/s).

## Results and discussion

### Uptake of Pd(II) from solution

Initial tests compared the rate of reduction of Pd(II) from Na_2_PdCl_4_ solutions using H_2_ or formate as electron donors following the initial biosorption of Pd(II). Under H_2_ complete removal of Pd(II) was invariably observed within 5–10 min whereas the removal of Pd(II) via formate required ~1 h. By substituting PdCl_2_ for Na_2_PdCl_4_ the rate of Pd(II) reduction was doubled, possibly attributable to a greater predominance of Pd^2+^ ions at the lower concentration of chloride, i.e. with less tendency to form neutral (PdCl_2_) or anionic (${\text{PdCl}}_{3}^{ - }$) species in solution. Since Pd(II) can behave in a similar way to Ni(II), it is possible that cellular uptake and trafficking mechanisms for Ni(II) may have facilitated uptake of Pd(II) into the cells although the route of Pd(II) uptake following initial biosorption, and the effect of Pd(II) ions, remain to be confirmed, along with any increased toxicity effects of the 10 mM Pd(II) as used in this study [previous work has generally used 2 mM Pd(II)].

### Biodeposition of Pd(0) on bacterial cells

**Fig. 1:**
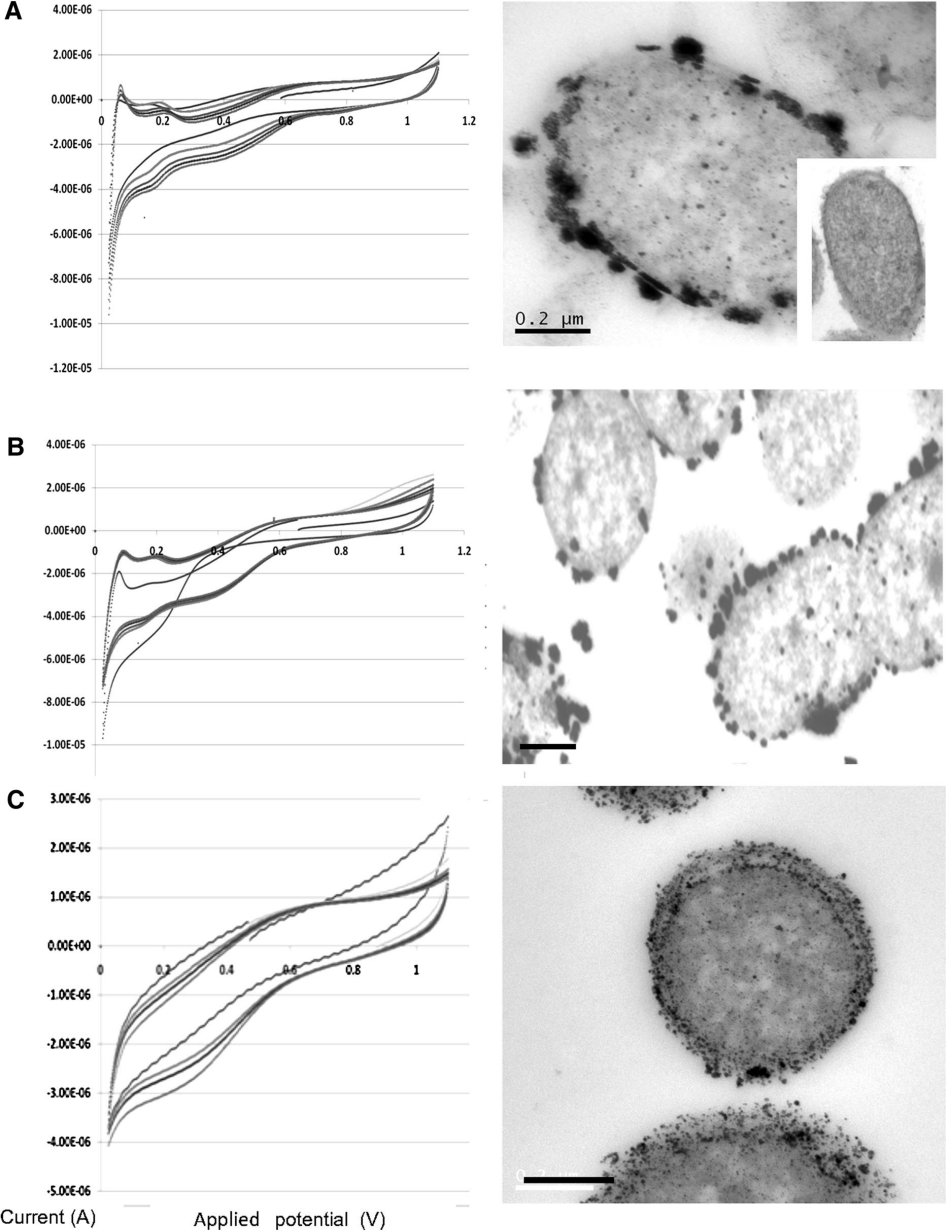
Formation of Pd-nanoparticles (20 wt%) on *E. coli* MC4100 using H_2_ and formate as electron donors (*right panels*) and cyclic voltammograms of the palladised cell preparations (*left panels*; multiple lines denote repeated scans). **a** H_2_ as electron donor for synthesis of Pd(0) from PdCl_2_ as the Pd(II) salt. *Inset* cells unchallenged with Pd(II). **b** H_2_ as electron donor for synthesis of Pd(0) from Na_2_PdCl_4_ as the Pd(II) salt. **c** Formate as electron donor for synthesis of Pd(0) from Na_2_PdCl_4_ as the Pd(II) salt. *Bars* 200 nm

**Fig. 2:**
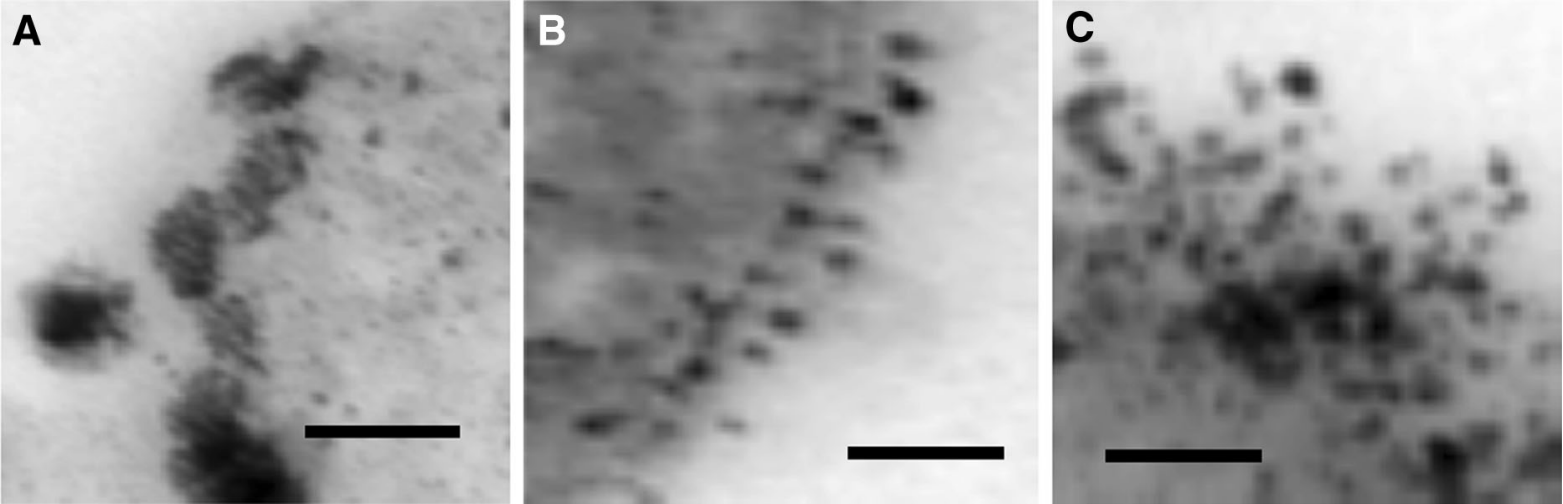
Detail of Pd-nanoparticles formed on the surface of *E. coli* at 20 wt% loading at the expense of **a** H_2_ + PdCl_2_; **b** formate + PdCl_2_ and **c** formate + Na_2_PdCl_4_. *Bar* 100 nm

Material made from PdCl_2_ under H_2_ showed large Pd-NPs (Fig. 1a; confirmed as Pd by EDS; not shown) not visible on Pd-unchallenged cells (inset). Some deposits showed co-localisation in the outer and inner membranes. A similar pattern of Pd-deposition was obtained using Na_2_PdCl_4_ (Fig. 1b). The Pd-NPs made under H_2_ were generally smaller when made using Na_2_PdCl_4_ (Fig. 1b) than with PdCl_2_ (Fig. 1a). Those made using formate were very small (Fig. 1c) and were indistinguishable with respect to the palladium salt used (Fig. 2b, c) but were more numerous than those made under H_2_ (Figs. 1, 2a). A closer examination of the deposited NPs (Fig. 2) reveals morphological differences. NPs made under H_2_ appear large but comprised, in some cases, agglomerations of small NPs, visible as separate entities (Fig. 2a). In contrast NPs made via formate were small and well separated (Fig. 2b, c).

We suggest that the use of formate is rate-limiting due to the need to split it into H_2_ and CO_2_ in order to reduce Pd(II) at the expense of generated H_2_. This can occur either autocatalytically by ‘seeds’ of Pd(0) that split formate catalytically, or enzymatically using formate H_2_ lyase (FHL). However, FHL is not a periplasmic enzyme, which is in contradiction to the localisation of most of the NPs seen by electron microscopy (Figs. 1c, 2b, c). FHL activity may be involved in intracellular Pd-NP deposition; some Pd-NP deposits are visible intracellularly as well as in association with the inner membrane (Fig. 1). If the Pd-NP synthesis reaction is limited by the rate of formate cleavage it is likely that some Pd(II) persists at the cell surface long enough for it to localise onto additional potential nucleation sites rather than rapid initial nucleation and consolidation onto fewer sites, nearer to the cell surface, when promoted by H in a fast reaction.

A detailed study of the roles of hydrogenases in the pattern of deposition of Pd(0) by *E. coli* was reported (Deplanche et al. 2010). Unlike *Desulfovibrio,* which has periplasmic hydrogenases involved in Pd(0) deposition (Mikheenko et al. 2008), those of *E. coli* are cytoplasmic membrane-bound, with the inward-facing hydrogenase 3 component of the FHL complex making cytoplasmic-facing Pd(0). Such an arrangement is visible on the inner membrane of the cells in Fig. 1c, confirmed elsewhere using a mutant which expressed only hydrogenase 3 (Deplanche et al. 2010). In contrast the inner membrane-localisation of Pd(0) when made under H_2_ evidenced more discrete, denser depositions (Fig. 1a). Deplanche et al. (2010) showed that there is no single hydrogenase involvement in Pd(0) manufacture by *E. coli*; several hydrogenases are involved, the size of the Pd-nanoparticles relating to the enzyme that produced them. A similar result was reported using *D. fructosovorans* (Mikheenko et al. 2008). Other work, in contrast to this study and using *S. oneidensis,* suggested that, with formate, Pd-NPs were larger (and fewer) than by using H_2_ (de Windt et al. 2005) but de Windt et al. (2006) also showed size control of Pd-NPs according to the conditions. Søbjerg et al. (2011) reported that NPs can be size-controlled by adjusting the biomass/Pd ratio while Williams (2016) showed, with bio-Pd_*D. desulfuricans*_, that, by using the same biomass (mg)/metal (total atoms) ratio, different NP sizes resulted according to whether a small volume of 10 mM Pd(II) was used (i.e. as in this study), or a fivefold more dilute solution (2 mM, as in other work). This suggests that metal toxicity (i.e. metal concentration) may play a role in determining the number of loci that go on to support NP growth if this is enzymatically-mediated. The involvement of other enzymes than hydrogenases is not precluded; hydrogenase-deficient mutants of *E. coli* made Pd(0) but this was localised as fewer, larger NPs on the cell surface (Deplanche et al. 2010).

### Electrochemical analysis of bio-Pd(0) made at the expense of H_2_ and formate

CV has been used to probe the crystal surface structure of bio-Pt. (Attard et al. 2012). Using bio-Pd made under H_2_ CV gave evidence for electrochemical activity of the bio-NPs in a native (non-sintered) state (Fig. 1a, b), attributed to the adsorption/desorption of protons, and indicating an electrochemically active surface area and so a routine test for fuel cell catalysts. In contrast bio-Pd prepared using formate as the electron donor gave well separated NPs, with the material showing no electrochemically active surface (Fig. 1c) and hence little potential for fuel cell use.

**Fig. 3:**
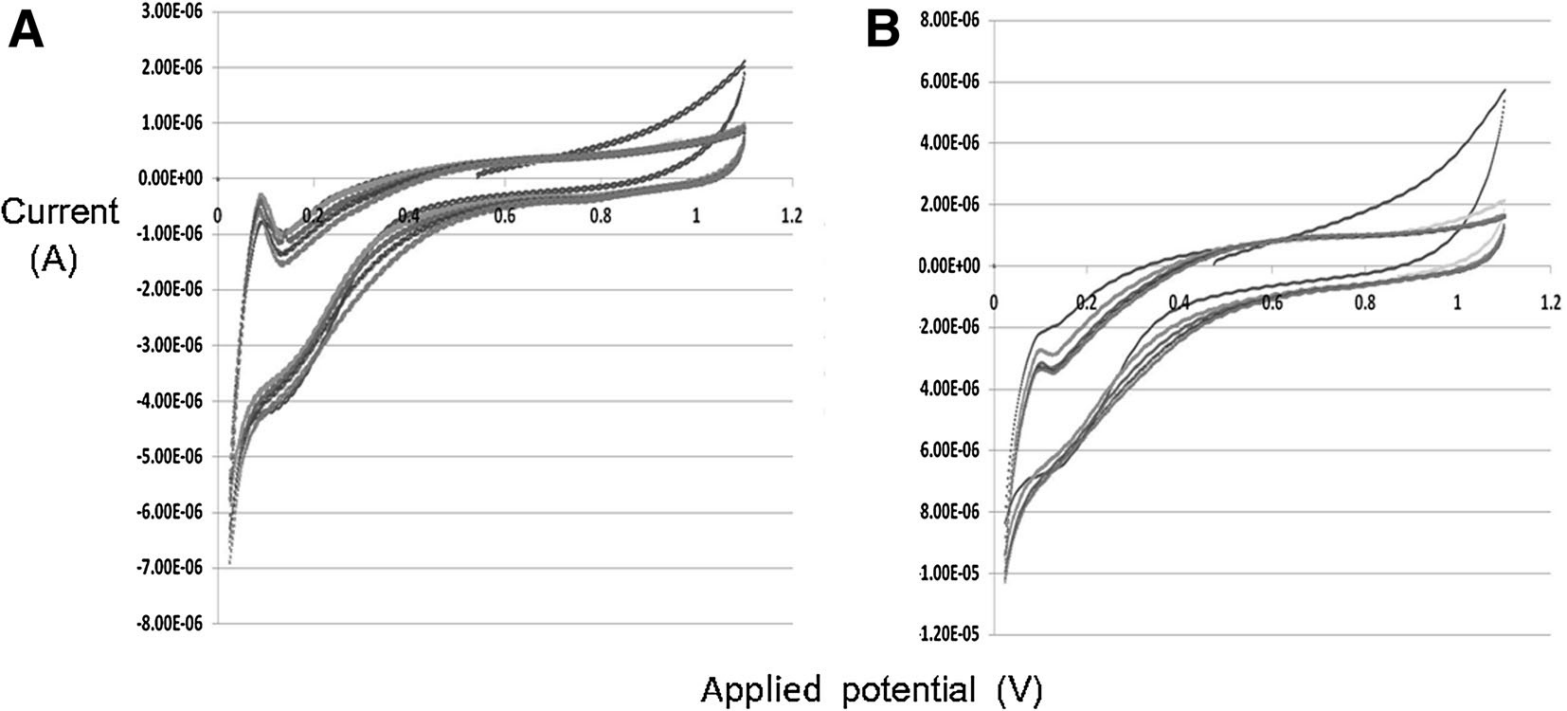
Cyclic voltammograms of cell preparations of bio-Pd on *S. oneidensis* made from PdCl_2_ at the expense of H_2_ (**a**) and formate (**b**) as described for *E. coli*

The effect of a hydride layer on the surface of bio-Pd must be considered; such a ‘masking’ layer underlied the choice of bio-Pt for detailed electrochemical studies (Attard et al. 2012). This phenomenon introduces an uncertainty as to whether interactions are taking place at the surface of the Pd crystals in a true catalytic process or as a result of hydride formation or H_2_ absorbed within the crystal structure of the Pd-NPs. Electrochemical analysis was used (with this caveat); although cyclic voltammograms (CVs) from samples loaded with 20 % (w/v) Pd(0) made under H_2_ exhibited electrochemically active areas proper quantification would require integration under the desorption peaks and corrections for scan rate and nominal charge per cm^2^ for the Pd. This is not trivial. However the response was observed regardless of the Pd salt used (Fig. 1a, b). Bio-Pd_*E*. *coli*_ also showed relatively small capacitance and resistance when compared to bio-Pd_*Shewanella oneidensis*_ (Fig. 3a). Comparing Figs. 1 and 3, it appears that the capacitance shown by the bio-Pd_*E coli*_ is ~0.5–0.6 microamps whereas that of bio-Pd_*S oneidensis*_ is ~twice this value. The resistance also appears slightly lower in bio-Pd_*E coli*_ due to the less sloping baselines to the voltammetry. Note that these are (approximate) observations made directly from the voltammetry and no separate measurements have been made to quantify them.

In contrast to CVs obtained from H_2_-derived bio-Pd (Figs. 1a, b, 3a), bio-Pd made using formate shows no significant electrochemical interaction using either cell type (Figs. 1c, 3b). The Pd-NPs made from formate were held apart by biomass materials (Fig. 2b, c); hence this study shows no intrinsic conductivity via biomass layers between the Pd-NPs of intact cells. In contrast bio-Pd made under H_2_ was electrically conductive, possibly attributed to direct contact between adjacent Pd-NPs. Wu et al. (2011) argue for a role of Pd-NPs in extracellular electron transfer with, at high Pd-loading, no requirement for cellular metabolism since Pd(0) splits H_2_ catalytically to yield electrons.

From Fig. 1a, b several conclusions can be inferred. The CVs show several ‘fingerprint’ peaks that are characteristic of proton adsorption occurring at the catalyst surface. As noted above the system used may lead to ambiguity due to the behaviour of protons towards Pd crystals. The possibility of proton absorption into the structures means the method cannot be used to assess conclusively the Pd-NP surface features (kinks, terraces etc.) or the electrochemical surface area as a true monolayer of protons may not be formed. However the method allows for comparison of the behaviour of Pd-NPs when compared between separate samples and it is notable that a difference in CV peaks was observed between bio-Pd(0) samples produced using formate or H_2_ and also Na_2_PdCl_4_ and PdCl_2_ as the palladium source, as well as between two species of bacteria that produced bio-Pd(0) at the expense of H_2_ from PdCl_2_ (Figs. 1a, 3a).

It is proposed that the reason underlying the observed differences between materials made using H_2_ or formate is the size and relative positioning of bio-NPs. With formate, deposition appears to be relatively uniform across the cross sectional area of the cell surface, producing relatively small, unaggregated NPs, whereas under H_2_ the deposition occurred preferentially at the cell membranes forming larger NPs (Fig. 1a, b) as aggregates (Fig. 2a). During electrochemical analysis the latter produced larger signals, with a much larger associated non-faradaic charge transfer. Nominally this indicates a higher surface area available for electrocatalytic reaction, however due to the size of the NPs it may be associated with a larger volume of proton adsorption within them.

A repeated voltage scan (e.g. from samples using H_2_ for bio-Pd manufacture from PdCl_2_; Figs. 1a, 3a) shows CVs recorded during repeated scans, each experiment representing several additional voltage cycles. There are several possible explanations for an observed change in the desorption peak at *E* ^0^ 0.05–0.2 V seen in Fig. 1a. It is possible that poisoning of the catalyst surface occurred via impurities in the electrolyte or compounds associated with the bacterial surface (note that such changes were also seen when experiments were repeated using different commercial sources of perchloric acid and alternative sources of distilled water). Other studies have confirmed using bio-Pt that even material ‘cleaned’ using NaOH contains a component of residual cellular material which, when removed via further chemical cleaning and then electrochemical removal of the final residua, leads to unmasking of various additional features in the CV from which information about the actual crystal surface can then be obtained (Attard et al. 2012). A similar analysis is not possible using bio-Pd due to the masking effect of the H_2_ chemistry at the catalyst surface (above). However, it could be hypothesised through analysis of the CVs that a 110 crystal surface, which initially produces the largest charge transfer, becomes poisoned and that the 100 surface increases in dominance. This may be enhanced by the 100 surface being electrochemically ‘cleaned’; such progressive cleaning results in loss of electrochemical resolution due to nanoparticle aggregation (Attard et al. 2012).

Yong et al. (2007) noted little activity of bio-Pt or bio-Pd per se as an oxidation catalyst in a PEM-FC anode and that sintering was required to carbonise the material in order to confer conductivity. In contrast the present study suggests that the biomass residua may, in fact, be electrically conductive given sufficient charge accumulation to overcome the ohmic resistance.
